# Pityriasis Amiantacea-Like Eruptions in Scalp: A Novel Manifestation of Pityriasis Rosea in a Child

**DOI:** 10.4103/0974-7753.77524

**Published:** 2010

**Authors:** Vijay Zawar

**Affiliations:** Consultant Dermatologist, Skin Diseases Center, Opp. Hotel Panchavati, Nashik, Maharashtra, India

**Keywords:** Atypical, pityriasis amantacea, pityrisiasis rosea, scalp

## Abstract

Unusual clinical features are known in pityriasis rosea (PR). We report a case of a child who presented with onset of PR in scalp, clinically mimicking pityriasis amiantacea. Careful clinical observations and follow-up led us to appropriate diagnosis.

## INTRODUCTION

Pityriasis rosea (PR) is self-limiting papulo-squamous disorder, which presents in its classical form as herald plaque followed by generalized eruptions on trunk in “Christmas-tree” pattern. Uncharacteristic morphology and or distribution in a case of PR refer to a category of ‘Atypical PR’. Atypical clinical presentation of PR are seen in approximately 20% of patients.[1,2] Pityriasis amiantacea (PA) is an inflammatory condition affecting scalp wherein there are thickly adherent, “asbestos-like” scales binding down tufts of proximal hairs.[3–5]

## CASE REPORT

**Figure 1:**
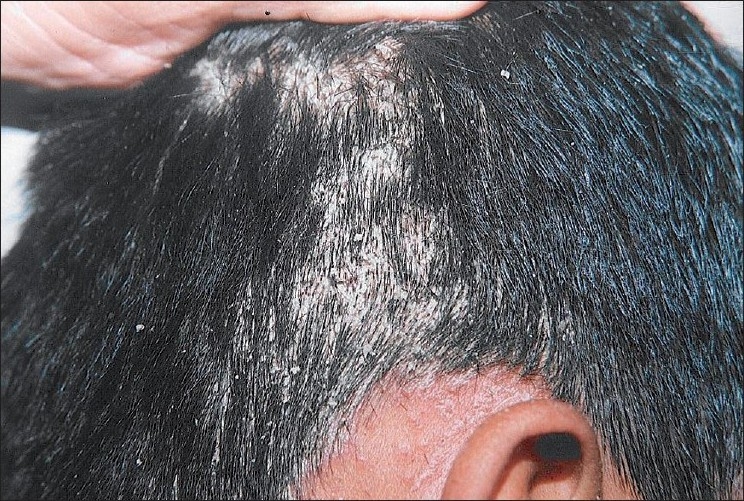
Thick, adherent “asbestos-like” scales with buried tufts of hairs on the scalp

**Figure 2:**
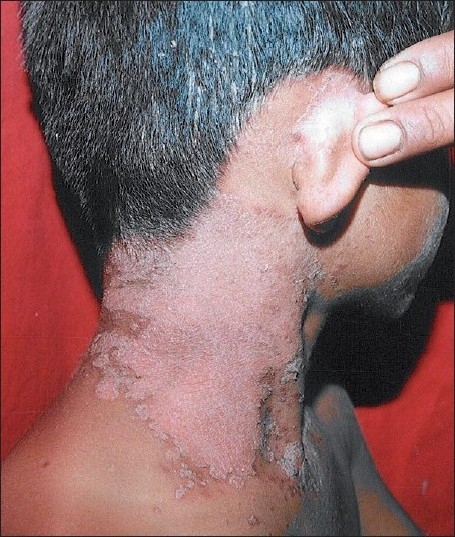
Sudden appearance of scaly sharply demarcated erythematous lesions on side of neck in the next week

**Figure 3:**
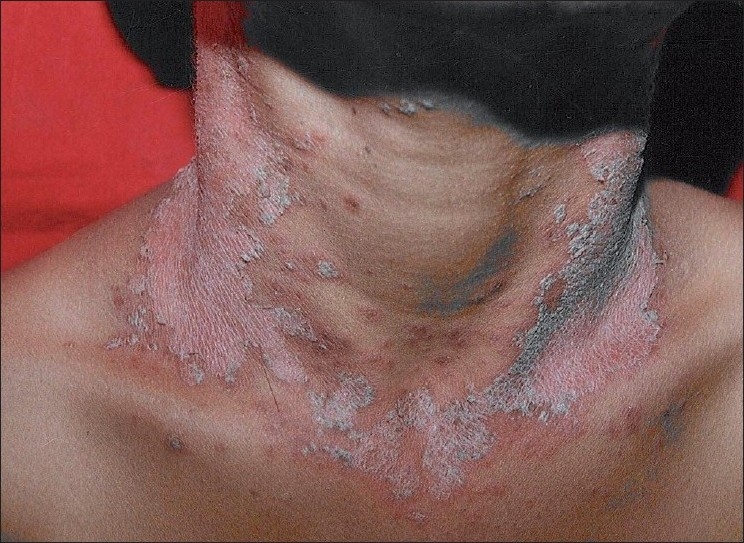
Sudden appearance of scaly sharply demarcated erythematous lesions on front of neck in the next week

**Figure 4:**
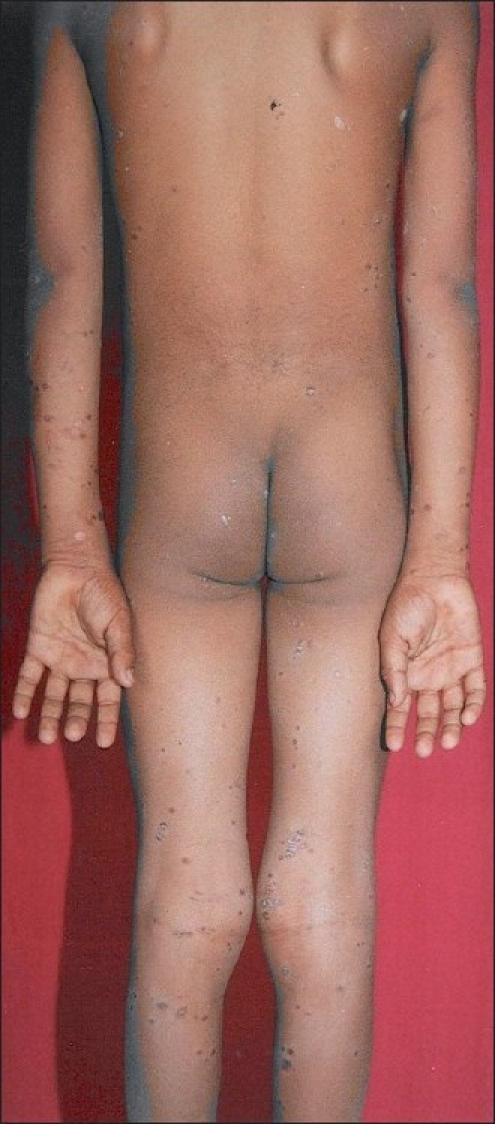
Bilateral, scaly sharply demarcated erythematous plaques with collarette scales at places on anterior trunk and upper extremities; few lesions follow Langer’s lines

**Figure 5:**
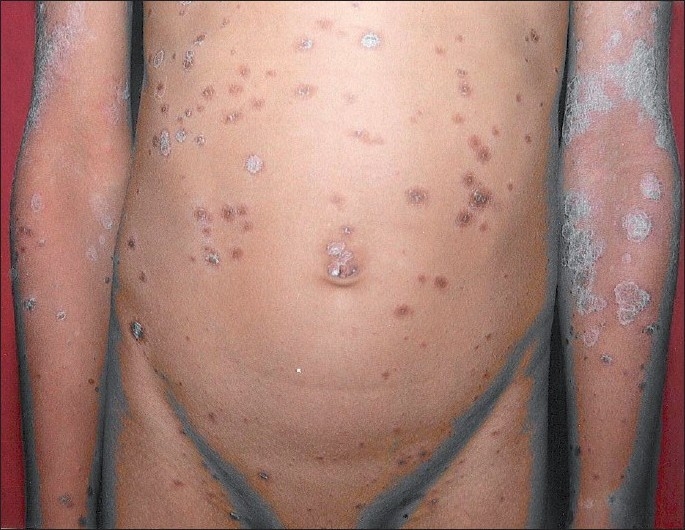
Bilateral scaly sharply demarcated erythematous lesions on posterior trunk and lower extremities; few lesions follow Langer’s lines and exhibited collarette scales

An otherwise healthy, 9-years-old male child was brought by parents for slightly pruritic, thick gray-white, adherent scaling in scalp of sudden onset, which rapidly progressed within a week [Figure 1]. This was followed by erythematous scaly conglomerated plaques on the back and front of neck and later spread to sides of neck and downward on the trunk gradually affecting upper and lower extremities over next 10 days [Figures 2–5]. Some of the lesions show collarette scales and seem following Langer’s lines. There was history of short-lasting upper respiratory infection presenting with coryza, sore throat and low-grade fever 1 week preceding the onset of scalp lesions. There was no treatment sought either for the respiratory infection or for the scalp eruptions. There was no history of similar illness or psoriasis, seborrheic dermatitis, alopecia areata and atopic dermatitis in past or family of this patient. There was no similar illness in this child before or in the family members. There was no history of scalp disease in particular. There was no history of any new topical application including shampoo, hair oil, soap or other cosmetic preparations on scalp before or after the onset of scalp eruptions.

On initial assessment, we made a tentative diagnosis of PA. As we ordered the investigations, the eruptions on neck and trunk appeared and we suspected a diagnosis of psoriasis but cutaneous lesions apart from scalp lacked typical silvery scales and Auspit’s sign was absent. Appearance of several smaller erythematous plaques with collarette scaling, few following Langer’s lines on trunk and extremities suggested a diagnosis of PR.

His baseline investigations including blood counts, urinalysis, random blood sugar level were normal. Skin scraping for KOH examination was negative for fungus and wood’s lamp examination did not show fluorescence. ASO titer and throat swab were negative. VDRL test and HIV antibodies were negative. Skin biopsy from scalp was declined but it was done from the truncal lesion, which revealed focal parakeratosis, epidermal spongiosis, extravasation of rbcs in upper dermis and mixed inflammatory infiltrate in dermis. There was no histological evidence of psoriasis.

We prescribed salicylic acid and coal tar containing shampoo preparation for scalp and vaseline for other eruptions on trunk and extremities and 5 mg desloratidine once daily for 10 days. The truncal lesions completely resolved with little post-inflammatory hypopigmentation, while the scalp lesions slowly but completely responded over two weeks. We followed the patient for next 2 years for recurrence or appearance of any relevant eruptions on the scalp or elsewhere on the body. However, the patient remained clear and healthy.

## DISCUSSION

Considering the morphological presentation of scalp, we were initially confused it to be PA. However, further close observations and investigations of subsequent eruptions led us to believe that PR was the most likely diagnosis.

PA is commonly seen in psoriasis, tinea capitis, alopecia, staphylococcal infection and lichen planus of scalp.[3–5] However, none of these were likely in our patient.

Classical areas of knee and elbows were uninvolved and Auspit’s sign was negative. Histologically, psoriasis is always distinctive. With sparing of retroauricular areas, eyebrows and paranasal areas and with lack of typical greasy scales, seborrheic dermatitis was unlikely. With rapid appearance of multiple bilateral eruptions on neck and trunk in a short span of time with no previous history of atopic dermatitis and no recurrence of lesion in future does not support likelihood of atopic eczema. Negative KOH study and absence of fluorescence in scalp ruled out tinea capitis, though fluorescence negative tinea capitis is known. Staphylococcal infection has been suggested to be a propagating factor for PA.[6] However, our patient responded without antimicrobial therapy.

In such situation, diagnostic criteria put forth by AAT Chuh[7] were very helpful while arriving to a near certain clinical diagnosis. Our patient fulfilled all essential criteria including discrete circular or oval lesions, scaling on most lesions and peripheral collarette scaling on two or more lesions. After having satisfied all the exclusion criteria, he fulfilled only one optional criterion that majority of the truncal lesions were along the ribs. However, the diagnostic criteria were immensely useful in differentiating the condition from several close differential diagnoses.

Indian children often present with atypical presentations of PR.[8,9] Scalp involvement in PR is unusual and is rarely reported in the literature. Amar *et al*.[10] indicated that PR particularly in black children has a different presentation and course than described in the textbooks. In their series of patients, 8% of patients had scalp involvement. PA-like presentation has not been described in PR to the best of our knowledge. We can not explain such appearance of lesions in scalp. It may be speculated that sudden onset in multiple pruritic eruptions in the scalp with conglomeration may have lead to such morphological presentation.

## CONCLUSIONS

PA-like presentation has not been described in context to PR in Indian literature. This was a distinct manifestation of PR having onset in scalp in our patient. This entity runs a risk of misdiagnosis and it must be differentiated from psoriasis and seborrheic dermatitis. Hence, careful observation and meticulous follow up is necessary. It is important that clinicians should be aware of such atypical presentation of PR. The case is being reported here for its rarity.
